# Complex Extreme Sea Levels Prediction Analysis: Karachi Coast Case Study

**DOI:** 10.3390/e22050549

**Published:** 2020-05-14

**Authors:** Faisal Ahmed Khan, Tariq Masood Ali Khan, Ali Najah Ahmed, Haitham Abdulmohsin Afan, Mohsen Sherif, Ahmed Sefelnasr, Ahmed El-Shafie

**Affiliations:** 1 Institute of Environmental Studies, University of Karachi, Karachi 75270, Pakistan; faisal_ahmad_khan@hotmail.com (F.A.K.); tariqmak@uok.edu.pk (T.M.A.K.); 2 Institute for Energy Infrastructure (IEI), Universiti Tenaga Nasional (UNITEN), Kajang 43000, Selangor, Malaysia; Mahfoodh@uniten.edu.my; 3 Institute of Research and Development, Duy Tan University, Da Nang 550000, Vietnam; 4 National Water Center, United Arab Emirates University, Al Ain P.O. Box 15551, UAE; MSherif@uaeu.ac.ae (M.S.); ahmed.sefelnasr@uaeu.ac.ae (A.S.); elshafie@um.edu.my (A.E.-S.); 5 Civil and Environmental Eng. Dept., College of Engineering, United Arab Emirates University, Al Ain 15551, UAE; 6 Department of Civil Engineering, University of Malaya, Kuala Lumpur 50603, Malaysia

**Keywords:** extreme sea level prediction, complex system prediction, annual maximum method, joint probability method, Pakistan coast

## Abstract

In this study, the analysis of the extreme sea level was carried out by using 10 years (2007–2016) of hourly tide gauge data of Karachi port station along the Pakistan coast. Observations revealed that the magnitudes of the tides usually exceeded the storm surges at this station. The main observation for this duration and the subsequent analysis showed that in June 2007 a tropical Cyclone “Yemyin” hit the Pakistan coast. The joint probability method (JPM) and the annual maximum method (AMM) were used for statistical analysis to find out the return periods of different extreme sea levels. According to the achieved results, the AMM and JPM methods erre compatible with each other for the Karachi coast and remained well within the range of 95% confidence. For the JPM method, the highest astronomical tide (HAT) of the Karachi coast was considered as the threshold and the sea levels above it were considered extreme sea levels. The 10 annual observed sea level maxima, in the recent past, showed an increasing trend for extreme sea levels. In the study period, the increment rates of 3.6 mm/year and 2.1 mm/year were observed for mean sea level and extreme sea level, respectively, along the Karachi coast. Tidal analysis, for the Karachi tide gauge data, showed less dependency of the extreme sea levels on the non-tidal residuals. By applying the Merrifield criteria of mean annual maximum water level ratio, it was found that the Karachi coast was tidally dominated and the non-tidal residual contribution was just 10%. The examination of the highest water level event (13 June 2014) during the study period, further favored the tidal dominance as compared to the non-tidal component along the Karachi coast.

## 1. Introduction

Many studies were made for extreme sea levels from several other regions, which helped the researchers conclude that, for tidally dominated coasts, most often the cause behind frequent extreme sea level events is sea level rise (SLR) [1,2,3,4]. Mean sea level trends, determined from the tide gauge data sets, were noticed as the main cause of the height increment of extreme sea levels [2,5,6,7]. A significant increase in the frequencies of extreme sea levels is expected with the global SLR [8,9].

The Arabian Sea and Bay of Bengal rim countries are very much prone to SLR, storm surges, high waves and swells [10,11,12]. Pakistan’s coastline values its aesthetic, ecological and economic assets. Sustainable coastal management of increasing population stress and the protection of coastal assets has become challenging for the concerned authorities. In the coming future, climate change is a severely intimidating issue for coastal management [12]. Because of the changing climate and SLR, the coast is not stagnant anymore and due attention is required from coastal planners and managers. For better coastal management it is necessary to assess the impact of sea level and climate changes. 

If there is a mean SLR then the frequency of extreme sea levels will also increase. Even a slight change in the mean sea level can have a major increasing impact on the frequency of extreme sea level events. This will intensify the effect of tropical cyclones [1,12], having more amplified storm surges hitting the coast and experiencing higher extreme sea levels. The impact of SLR is expected to be intensified due to increased meteorological events, by shortening the return periods of extreme sea levels.

Sea level analysis with reference to extreme events is very much needed because there is not much evidence available of any such studies conducted for the Karachi coast in the recent past. Extreme sea level events can be predicted and visualized by studying their association with tidal fluctuations and meteorological extreme events [13]. All the components related to the observed sea level, including mean water level, surges and tides, should be considered for the reliable estimation of future maximum water levels [14]. The co-occurrence of high tides and extreme meteorological events make a coast vulnerable to coastal flooding [15]. Coasts with abrupt increasing mean sea level trends must be investigated for extreme sea levels in upcoming years for the provision of technical bases for proper coastal management [16]. As far as coastal management is concerned, the variability of extreme sea levels is of much concern with reference to climate changes in the area. To estimate the return period and extent of maximum water levels, observed sea level data from tide-gauges provided potent bases for researchers [17].

Erosion is one of the known impacts of SLR, which furthermore is a reason for coastal slumping. The Bruun rule can estimate the extent of coastal slumping caused by SLR [5,18,19,20]. According to the Bruun rule, the rate of coastal slumping is 100 times the extent of SLR. This multiplied impact will have far-reaching consequences for the coastal infrastructure. In addition, the accelerated erosion of the coastline, the groundwater contamination and seawater intrusion are the direct consequences of SLR and the associated extreme events [12,21,22].

Statistical examination of the observed sea level is a common technique to predict extreme sea levels along with their return periods. Many statistical and mathematical approaches, like the annual maximum method (AMM), peak over threshold (POT) method and the joint probability method (JPM), have been used in the past. The accuracy and reliability of any used technique for predicting extreme sea levels depend on the frequency and length of available observed sea data. In the present study, we used AMM and JPM to analyze 10 years of hourly sea level data.

The AMM is a useful tool to estimate the return periods by calculating the probabilities of any extreme event. It has been shown that the annual maxima exhibited an almost linear increase when plotted against the logarithm of the number of years, hence it could be a convenient estimator for the recurrence of extreme events [23]. 

The AMM involves the analysis of a series of annual maxima to predict extreme sea levels for the future. Since this method uses only one value per year, the length of available data is very important. This method can be used by considering the limits for prediction [24]. Pugh and Vassie presented the JPM in the year 1978. In comparison, the JPM can be considered as a reliable technique even for short-term data and can be used for long-term predictions [25].

The AMM is based on an analysis of frequency, per year, of a time series data set. The quality of data should therefore be ensured in terms of the possibility of any outlying value. The main limitation of the AMM is the continuity of the data record. Split data records are not reliable for good estimation [26]. For the AMM the estimation of the return period of any extreme event can be done for the number of years that is five times the available number of years of data [24]. The JPM, being more complex, relies on the availability of hourly sea level data. Detailed examination of sea level oscillation is only possible with high-frequency data. Although the length of data can be compromised to a few years, the execution of the method needs not only the tide gauge data but also the predicted tide level data for each hour. In terms of the accuracy of estimation of the return periods of an extreme event, the JPM could be considered as more reliable than the AMM [27].

## 2. Study Area 

**Figure 1:**
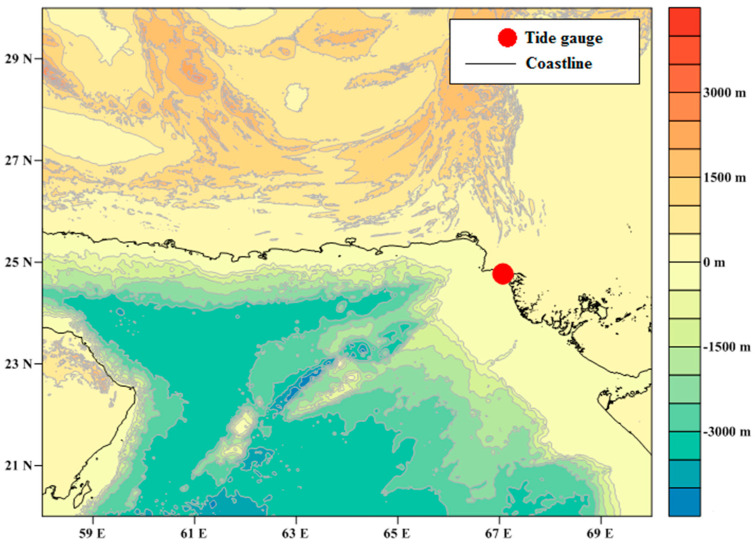
Bathymetry, topography and the location of the tide gauge at Karachi port, along the Pakistan coast.

The Pakistan coast has semi-diurnal tides with diurnal inequality. The mean tidal range is 2.3 m for the Karachi port. The proposed study comprised the analysis for extreme events linked with strong storm surges and extreme high tides as impacts of rising sea levels in the Karachi coast. The extreme sea level analysis, for the assessment of the return periods of different extreme sea levels, is very beneficial for effective coastal management and provides technical bases for coastal engineers [28]. In this extreme sea level study, the estimation of return periods was done by analyzing the hourly tide gauge data recorded at Karachi port along the Pakistan coast. The tide gauge was installed at the north side of the slipway control room at Karachi port trust at 24.8° N and 66.9° E. For the sea level measurement, the chart datum was 4.39 m below a benchmark, which was marked 0.5 m above ground level. The bathymetry, topography and the location of the tide gauge at Karachi port is shown in Figure 1. The study will be very useful in the identification of the issues associated with extreme sea level events and the possible effects of a rising sea level on the low lying areas along the coastal belt of Pakistan, such as the Indus deltaic region and Karachi coast.

A range of natural hazards poses risks to the coastline of Pakistan. Extreme sea level events, by SLR and storm surges, can initiate coastal flooding in the low lying regions along the coast. The shorelines may experience severe erosion during extreme sea level events. Saltwater intrusion is also a very serious problem in the low lying Indus delta. This problem of seawater intrusion has been reported by many authors [12,21,22,29,30,31,32,33]. 

In the coming future, the intensity and frequency of extreme sea levels are expected to increase due to the intensified impact of weather conditions and mean SLR. Pakistan’s coastal territory is facing serious issues of coastal flooding and coastal erosion as impacts of the SLR. The observed tide gauge data at Karachi port indicated that the sea level is rising for the coast [34]. In the Karachi coast, the SLR was estimated at the rate of approximately 1.1 mm per year in the past, calculated in year 1988. The observed change in sea level may be treated as a eustatic rise, as the Indus delta is on a passive continental margin and isostatically stable [35].

The change in sea level is important for coastal morphology (i.e., erosion or accretion problems) and coastal inundation in the coastal areas of Pakistan. The Pakistan coast, being tidally dominated, is facing changes in the coastline due to the impact of high tidal activity and extreme sea level events. A limited number of studies have been conducted for coastal erosion in Pakistan. In the year 1997, the space and upper atmosphere research commission (SUPARCO), the national space agency of the government of Pakistan, conducted a study on the coastal erosion of Pakistan. The study concluded that in the Indus delta, accretion and erosion were observed during the period from 1978 to 1994 [22].

**Figure 2:**
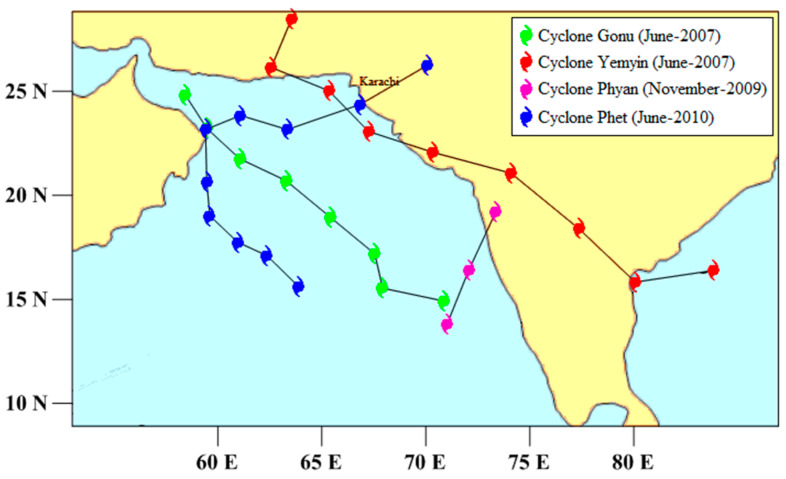
Tracks of all four of the tropical cyclones during the study period (2007 to 2016).

Storm surges can contribute to high sea levels, especially when accompanied by high tides. For studying extreme sea levels, meteorological forcing in terms of storm surges should also be considered [36]. During the study period, four tropical cyclones passed along the Pakistan coast (Table 1, Figure 2). In all of these four cyclones, Cyclone Yemyin was the deadliest as it killed 730 people in Pakistan and prominently contributed to the high tide gauge readings at Karachi port. 

## 3. Methodology

The tide gauge data of Karachi were analyzed for extreme sea levels. Research quality hourly tide gauge data were obtained from the University of Hawaii Sea Level Center (UHSLC) [37]. To calculate the return periods of extreme sea levels, a statistical investigation was done by using the AMM and JPM. To ensure the quality of the data, in terms of any outlying value, only “research quality” data were used. 

To calculate the return period of extreme sea level for the Karachi coast, harmonic and statistical analyses of the hourly observed sea level data for the past 10 years (2007–2016) were performed. In the first phase of the study, the hourly tide gauge data were decomposed into two components, i.e., the tide level and the surge (non-tidal component) for the whole time series, using an application WORLD TIDES [38] on MathWorks MATLAB v9.1 (Natick, MA, USA). The WORLD TIDES was used to examine the observed sea level data by using the harmonic analysis method of least squares (HAMELS). Power spectral density (PSD) calculation was a reliable analytical approach for analyzing the tide gauge data series [39]. The spectral analysis of the observed sea level data was done to calculate the PSD for the different frequencies, using the ‘pwelch’ function available in MATLAB. The 35 tidal constituents were used for tidal predictive analysis. The predicted tide data by WORLD TIDES were compared with the locally predicted tide tables generated by the Pakistan Navy Hydrography Department and were found to be reliable for use in the study. 

Generalized Pareto distribution (GPD) and POT are analytical approaches used by researchers for analyzing extreme sea levels. GPD is a probability distribution which explains the extreme value data in terms of scale and shape factors. The POT method works with data only above a specified data value, called the threshold. In comparison, the JPM counts on even short time series available data by considering the components of each data point and estimates the return periods of extreme sea levels with better precision.

For statistical analysis, the AMM and JPM were used. From the past 10 years of data, 10 annual maxima of observed sea levels were selected for analysis in the AMM [40]. The frequency analysis of the selected annual maxima was done and the probability P_η_ (in percent) Equation (1) and the return periods T_η_ (in years) Equation (2) for each sea level (η) were calculated. Following the formulae used for calculations:

(1) $$P_{\eta }=\frac{100\left(2r-1\right)}{2N}\;(\%)$$

where ‘r’ is the allocated rank of the annual maximum value and ‘N’ is the total number of values used. 

Then T(η) is calculated as the reciprocal of probability as

(2) $$T_{\eta }=\frac{1}{P\left(\eta \right)}$$

Return sea levels for the Karachi coast were to be determined after plotting a graph between the corresponding sea level ‘η’ and ‘lnT_η_’ and extending the plotline using a regression analysis. The selection of AMM for a small data set (i.e., 10 years) was considered with the limitation of the number of years for extrapolation. While using the AMM extrapolation of return periods, the result, in years, must be less than the magnitude of the product of integer five with the number of years for which the data is available [24]. Therefore, the return sea levels for the Karachi coast were to be determined for 50 years.

For the JPM, the joint probability of the tide and surge time series data was analyzed and the return periods were calculated. Initially, the hourly observed sea level data (η_O_(t)) were separated into tidal (η_T_(t)) and surge components (η_S_(t)) using the MATLAB application WORLD TIDES. 

Here:

η_o_(t) = η_T_(t) + η_s_(t) (3)

η_S_(t) = η_O_(t) − η_T_(t) (4)

By using the probabilities of tide P_T_(η_T_) and surge P_S_(η_S_), the joint probability of tide and surge P_J_(η) Equation (5) is determined [25,27]:

(5) $$P_{J}(\eta )=\int_{-\infty }^{\infty }\mathrm{PT}\left(\eta T\right)\times \;\mathrm{PS}\left(\eta S\right)\;d\eta S$$

Then, the return period T(η) for each sea level η is calculated and the return sea levels for the period of 50, 100 and 1000 years for the Karachi coast are determined. In this study, an episode of storm surge was discussed for the duration when Cyclone Yemyin hit the Baluchistan coast in June 2007.

## 4. Results and Discussion

**Figure 3:**
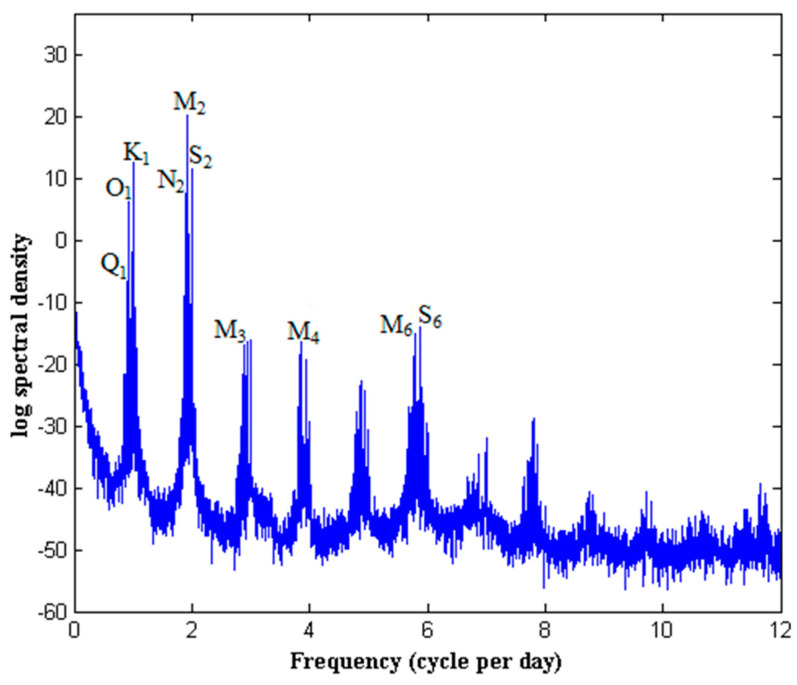
Spectral density of the tide gauge data.

Initially, the hourly tide gauge data was decomposed into two components i.e., the tidal levels and the surges. The contribution of surges to the observed sea level was calculated by subtracting the predicted tide levels from the tide gauge data. The spectral analysis of the Karachi tide gauge data, from 2007 to 2016, showed an almost inverse relation between the spectral densities and frequencies. In the PSD plot, many sharp peaks were present in low frequencies (Figure 3). In the analysis, the most prominent peaks were observed at 1.932, 1.003, 2, 1.895, 0.93 and 0.839cpd with periods of 12.42, 23.93, 12, 12.66, 25.81 and 26.87 h, referring to the tidal constituents M_2_, K_1_, S_2_, N_2_, O_1_ and Q_1_, respectively. The peaks present in the PSD facilitated decomposing the observed sea level data into surge and tide components. 

**Figure 4:**
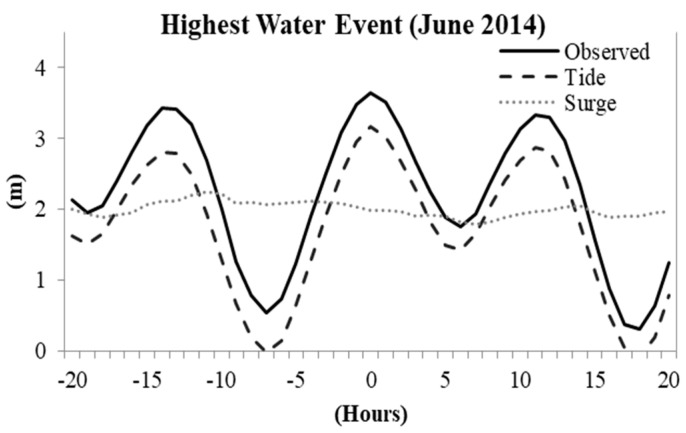
Hours from the highest water event at 11:00 PST (local time) on 13 June 2014 in the Karachi coast during the study period (i.e., years 2007–2016).

During the study period (i.e., years 2007 to 2016) the highest water level recorded was on 13 June 2014 (Figure 4). On this extreme event, the observed sea level was 3.64 m, dominantly contributed to by a tidal component of 3.17 m with a non-tidal residual of 0.47 m. Furthermore, the whole time series of the annual maximum along with the associated tidal level and non-tidal residuals showed that the Karachi coast was tidally dominated. 

On a global scale, the regions where the tidal contribution in the mean annual maximum water level is dominated, can be identified by calculating the ratio ‘γ’ [41]:

(6) $$\gamma =\bar{h}/(2.5\;\sigma_{p})$$

where $\bar{h}$ is the mean annual maximum water level and σp is the standard deviation of the predicted tidal elevation. The γ calculated for the Karachi coast was 1.1, which was showing the tidally dominated nature of the coast along with a 10% contribution of non-tidal residuals.

**Figure 5:**
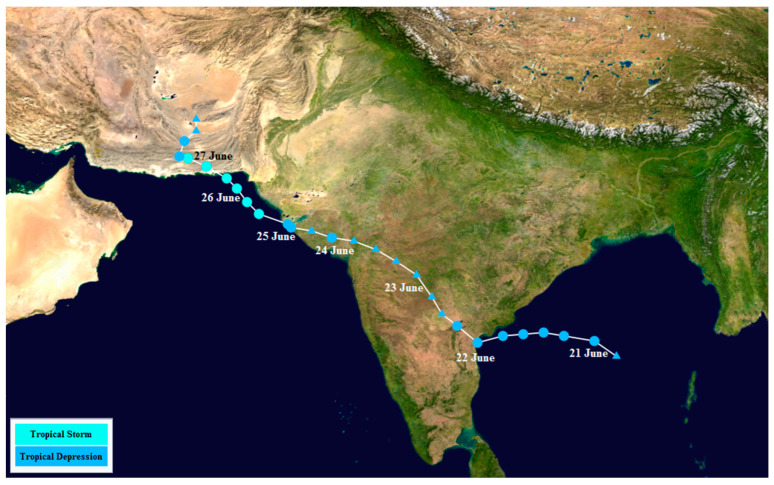
The track of Cyclone “Yemyin”, June 2007.

**Figure 6:**
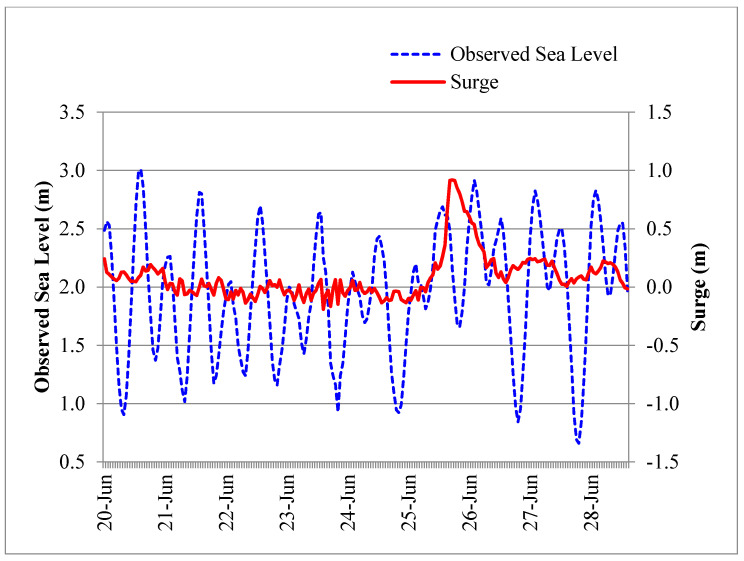
The signatures of the storm surges are seen in the tidal analysis of the Karachi Data during the period 20–28 June 2007 (Cyclone Yemyin).

During the study period, all the recorded meteorological events which were documented by the Pakistan Meteorological Department (PMD) were analyzed for studying the storm surge events. Four tropical cyclones, including Cyclone Gonu (June 2007), Cyclone Yemyin (June 2007), Cyclone Phyan (November 2009) and Cyclone Phet (May 2010) moved over the Pakistan coast. Cyclone “Yemyin”, being the most prominent of all four, crossed the Pakistan coast in the west of Karachi on 26 June 2007 (Figure 5). The signature of the storm surge event is seen in the tidal analysis of Karachi coast sea level data (Figure 6).

**Figure 7:**
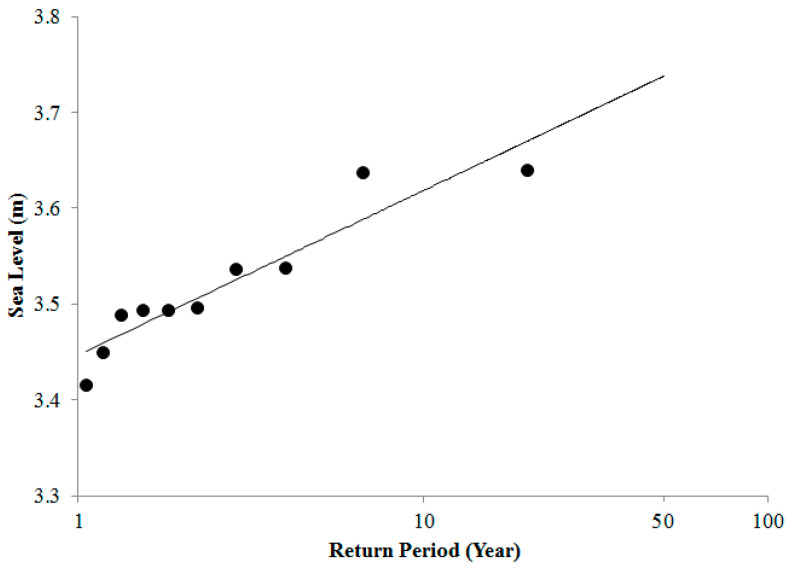
Return period by using the AMM (annual maximum method).

For extreme value analysis, the use of the AMM is common. In the AMM, the data set of each year is examined for the highest value of the year, which is taken as the annual maximum. The trend analysis of the maximum observed sea levels for each year, presented in Table 1, showed an annual increase of 2.1 mm for extreme sea levels. All the annual maxima for all the years in the data series were plotted against log10 of return periods to have a linear regression analysis. Then, the estimation of the extreme sea level for 50 years was done using the regression line. The probability and return periods are shown in Table 2 and Figure 7, respectively. 

**Figure 8:**
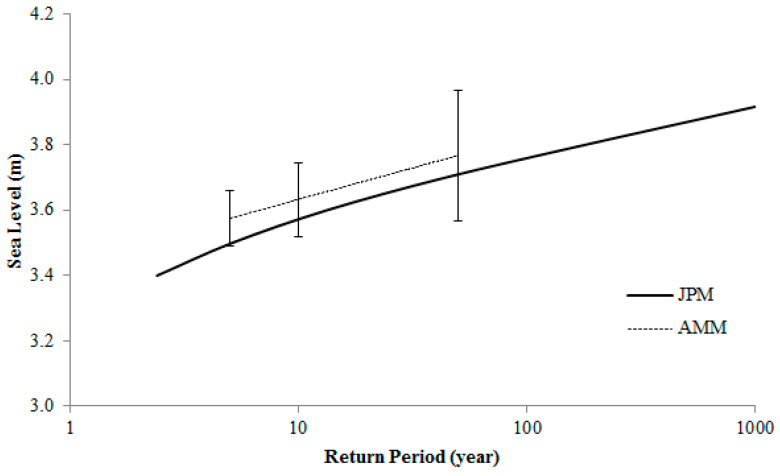
Estimated return period of extreme sea level utilizing the AMM and the JPM (joint probability method) for Karachi, Pakistan. The vertical lines show 95% confidence limits.

The predicted tide levels for each hour were deducted from the observed hourly sea levels and the residuals were taken as surges. As a result, from the observed sea level data, we obtained an hourly data series of the tides and the surges. The JPM provided good statistical grounds by considering the tides–surge interaction, for estimating the return periods of extreme sea levels. Based on the JPM and AMM approaches, the estimated return periods for the extreme sea levels for Karachi are shown in Figure 6. The determination of the 95% confidence was achieved to check the consistency of both methods (Figure 8). It could be observed that the consistent levels of confidence were attained using both the AMM and JPM approaches, which were more than the 95% confidence limits.

The summary of the return sea level height for 50, 100 and 1000 return periods by using the AMM and JPM are shown in Table 3.

**Figure 9:**
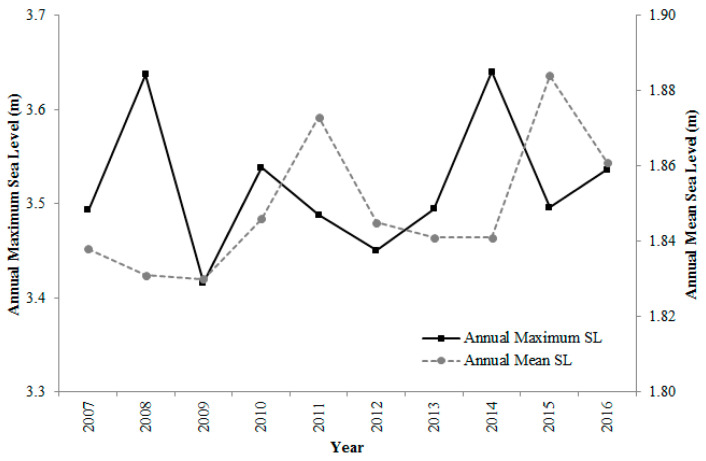
Annual mean and extreme sea levels for the Karachi tide gauge data from the year 2007 to 2016.

In comparison, both the rate of change of extreme sea levels and the rate of change of mean sea levels are showing an increasing trend. Observed sea level data at Karachi port for the study period i.e., 2007–2016, showed an increment rate of 3.6 mm/year for the mean sea levels and 2.1 mm/year for the extreme sea levels (Figure 9). Here at Karachi port, the SLR is contributing to increasing extreme sea levels along the Pakistan coast. The SLR may therefore increase the frequency of extreme sea level events with a subsequent decrease in their return periods.

## 5. Conclusions

The JPM and AMM provided useful statistical bases for the calculation of extreme sea levels along the Pakistan coast. These statistical techniques were used for the first time in the assessment of extreme sea levels for the Karachi tide gauge data. The present study showed the prominent contribution of the tidal components in contrast with the non-tidal components of observed sea levels, for the estimation of extreme sea levels.

The extreme sea level analysis, presented in this study for the estimation of return periods, indicated the technical causes of a maximum water level. Although the results based on statistical bases should be rectified on the bases of coastal engineering, understanding meteorological events and more advance tidal observation will facilitate the outcomes of this study. 

The main problem during this study was the less and non-consecutive availability of tide gauge data. Furthermore, Karachi is the only station that has relatively long historical tidal data. It was suggested to install more tide gauges in the Sindh and Balochistan coasts. Thus, tide gauge installation along the whole Pakistan coast at different locations would help to analyze the tidal heights. Moreover, in the stations where tide gauges are not installed, the use of the satellite altimetry sea surface height data and numerical model results may probably help in the assessment of return periods of extreme sea levels [28,41].

Along the Pakistan coast at Karachi port, the intensification of coastal slumping due to SLR is putting the natural coastal defense at risk. Tidal height will increase due to SLR, which most probably causes coastal slumping [12]. Likewise, on the west side of Karachi on different sandy coastlines, erosion has been observed [42]. The erosion is taking place due to intensive wave action, seawater flooding and intensified tidal activity.

Increasing rates of mean sea levels are among the leading causes of more frequent extreme sea level events. Low lying regions of Indus are at risk due to SLR and extreme sea level events. In the next few decades, a minor SLR could cause significant suffering for the masses near the coast in this region. Coastal erosion is associated with the flooding of low lying areas and freshwater depletion in the Indus River due to seawater intrusion, which negatively affects mangroves, aquatic species and a variety of flora and fauna in the region. Therefore, if this ecological destruction is not considered, then the biological identity of the Sindh coast, including Karachi, is in danger as different aquatic species are reducing and many of them could disappear forever.

The present study can be used to project future SLR based on the present and past trends and the study of physical phenomena such as seawater intrusion and coastal erosion. Moreover, the knowledge of the present trends of SLR and extreme sea levels along the Karachi coast helps to adopt protective measures by following the estimated SLR and extreme sea level events, which usually have a great impact on the coastal community and ecosystem of the area. 

## Figures and Tables

**Table 1:** List of tropical cyclones during the study period (2007 to 2016).

Cyclone	Year	Dates
Cyclone Gonu	2007	1 June–8 June
Cyclone Yemyin	2007	21 June–26 June
Cyclone Phyan	2009	9 November–11 November
Cyclone Phet	2010	31 May–7 June

**Table 2:** The estimated value of the probability and the return periods by using the annual maximum method (AMM).

Rank r	Year	Annual Maxima (m)	Probability *p* = 100(2r-1)/2N (%)	Return Period T = 1/p (year)
7	2007	3.493	65	1.538
2	2008	3.637	15	6.667
10	2009	3.416	95	1.053
3	2010	3.538	25	4.000
8	2011	3.488	75	1.333
9	2012	3.450	85	1.176
6	2013	3.494	55	1.818
1	2014	3.640	5	20.000
5	2015	3.496	45	2.222
4	2016	3.536	35	2.857

**Table 3:** Summary of the return level estimation for 50, 100 and 1000 return periods.

Method	Return Level for 50 Year Period (m)	Return Level for 100 Year Period (m)	Return Level for 1000 Year Period (m)
Annual Maximum	3.74	-	-
Joint Probability	3.73	3.76	3.92
